# A 1-month ketogenic diet in patients with migraine gives a clinical beneficial effect associated with increased latency of somatosensory thalamo-cortical activity

**DOI:** 10.1016/j.cnp.2024.11.002

**Published:** 2024-11-15

**Authors:** Chiara Abagnale, Gabriele Sebastianelli, Francesco Casillo, Antonio Di Renzo, Vincenzo Parisi, Ettore Cioffi, Mariano Serrao, Jean Schoenen, Gianluca Coppola, Cherubino Di Lorenzo

**Affiliations:** a Department of Medico-Surgical Sciences and Biotechnologies, Sapienza University of Rome Polo Pontino ICOT, Latina, Italy; b IRCCS – Fondazione Bietti, Rome, Italy; c University of Liège, CHU Sart Tilman-B23, Giga-Neurosciences, Neuroanantomy, Liège, Belgium

**Keywords:** Thalamocortical network, GABA, Oscillations, Latency, Ketones

## Abstract

**Objective:**

Since the habituation deficit of evoked potentials could be related to abnormal thalamocortical drive, we searched for a modulatory effect of ketogenic diet (KD) on somatosensory-evoked thalamo-cortical activity. KD is effective in preventing migraine. Previous studies showed that KD normalises habituation of somatosensory and visual cortical evoked responses in parallel with a decrease in of migraine attack frequency.

**Methods:**

We electrically stimulated the median nerve at the wrist to record somatosensory high-frequency oscillations (HFOs) in twenty patients with episodic migraine with and without aura before and after one month of normo- (n = 9) or hypocaloric KD (n = 11). For pre-synaptic thalamocortical and post-synaptic cortical HFOs, we measured the latency of the negative oscillatory maximum, the intra-burst frequency, the number of negative peaks, and the maximum peak-to-peak amplitude.

**Results:**

In the total group of patients, the one-month KD significantly increased the latency of the negative oscillatory maximum in pre-synaptic, i.e. thalamocortical activity (t = 2.70, p = 0.015) and in post-synaptic HFOs, i.e. cortical activity (t = 3.08, p = 0.006). This effect could be attributed to hypo-caloric KD, as it was not found after normo-caloric KD. Other HFO parameters, such as amplitude, duration, or number of oscillations, were not affected.

**Conclusions:**

A 1-month hypo-caloric KD is able to delay the propagation of neuronal activity through the thalamo-cortical network. This effect does not seem to be correlated with the therapeutic efficacy of KD, but rather to low-calorie intake.

**Significance:**

Our results imply that consuming a restricted amount of calories could alter the balance between central excitation and inhibition in migraine.

## Highlights

• Unlike a normocaloric ketogenic diet, a 1-month hypocaloric KD delayed thalamocortical and cortical somatosensory high-frequency oscillations in migraine.
• This effect could thus be due to the low-calorie intake that may increase central GABA neurotransmission.
• This effect does not seem to be correlated with the therapeutic efficacy of KD on headache.

## 1 Introduction

Ketogenic diet (KD), due to the reduced carbohydrate intake, forces the body to shift from the glycolytic to the lipid beta-oxidative pathway of energy metabolism (Di Lorenzo et al. 2021). As a consequence, acetyl-CoA for the Krebs cycle is produced from ketones (acetoacetate, beta-hydroxybutyric acid and acetone) arising from free fatty acid metabolism (Paoli et al. 2013).

The standard KD is characterized by low carbohydrate and high fat intake. However, among the various KD protocols the very low-energy ketogenic diet (VLEKD), formerly also called very low-calorie KD (VLCKD) (Conte et al., 2023, Tagliabue et al., 2024), mimics a physiological state of prolonged fasting with protein sparing (Di Lorenzo et al. 2021). VLEKD was made popular to manage metabolic disorders and achieve weight loss (Atkinson and Kaiser, 1985, Moreno et al., 2014, Goday et al., 2016).

One of the numerous pathological conditions for which KD was shown to have a positive clinical outcome is migraine (Di Lorenzo et al. 2021). In a double-blind study Di Lorenzo et al., found that a 4-week VLEKD was significantly more effective than a very low-calorie non-ketogenic diet for migraine prevention, while causing similar weight loss and glycemic profile (Di Lorenzo et al. 2019b). Certain normocaloric KD protocols were also reported to be effective in migraine prevention (Tereshko et al. 2023b, 2023a).

Neurophysiological studies have been performed to gain a better understanding of how KD improves migraine. In patients with episodic migraine, a 4-week VLEKD normalized habituation of cortical somatosensory (SSEP) and visual evoked potentials (VEP) (Di Lorenzo et al. 2016). Another study from our group revealed that habituation of trigeminal pain-related cortical evoked potentials normalized during KD, while habituation of the nociceptive blink reflex, based on a brainstem circuit, remained deficient (Di Lorenzo et al. 2019a).

These studies suggest that KD can normalize evoked responses generated in the cortex but does not influence brainstem activities. We questioned therefore if it could have an additional modulatory effect on the ascending thalamocortical drive.

To answer this question, we explored in patients with episodic migraine the effect of a 1-month KD on SSEP high-frequency oscillations (HFOs) of which the early and late components reflect respectively afferent presynaptic thalamocortical and postsynaptic cortical activity.

## 2 Methods

### 2.1 Subjects

We recruited 20 patients with episodic migraine (15 without aura [MO, ICHD-III code 1.1], 5 with aura [MA, ICHD-III code 1.2.1]) aged 19–54 years. At the screening visit, we collected headache diaries covering the previous two months in order to gather data on clinical features, including mean monthly attack frequency (N), mean attack duration (hours). Nine patients had normal weight (BMI < 25) and 11 overweight (BMI ≥ 25, mean 28.6).

Being free of attacks for a minimum of three days prior to and following the recording sessions was a crucial requirement for inclusion, as checked by collecting the diaries and by post-recording telephone interviews. Regular medication intake (i.e. antibiotics, corticosteroids, antidepressants, benzodiazepines, preventive migraine medications, etc.) was an exclusion criterion, except for contraceptive pills. A history of any other kind of primary or secondary headache or any other neurological condition, systemic hypertension, diabetes, or other metabolic, autoimmune, or connective tissue diseases, and neuro-ophthalmological diseases, as determined by a thorough neuro-ophthalmological examination that included a visual acuity test, an intraocular pressure measurement, and indirect ophthalmoscopy, were additional exclusion criteria. All study participants received a complete explanation of the study and provided informed consent but were unaware of the precise electrophysiological outcome measure. The project was approved by the local Ethics Committee (Prot. n. 0097166/2021 of 12/05/2021).

### 2.2 Ketogenic diet

The total number of calories in the KD was assigned according to the BMI of the patients.

Overweight patients (BMI > 25) received a VLEKD with a total of 600 – 800 kcal, according to the European Food Safety Authority (EFSA) scientific opinion (EFSA NDA Panel, 2015). Normal-weight (BMI < 25) patients received a normo-caloric KD, according to their needs.

Overweight patients received 4-week VLEKD diet (≤800 kcal) with low carbohydrate (about 30 g/day carbohydrates), low fat (fixed 15 g lipids), and normal protein content (1.0–1.2 g/kg of desired weight proteins). This comprised five daily meals consisting of four protein shakes developed ad hoc as a protein supplement for VLEKD, one daily meal of meat (up to 200 g) or fish (up to 350 g) accompanied by salad and nutraceutical supplements. Normal-weight patients (BMI < 25) were administered a normo-caloric KD with low carbohydrate (about 15 g/day), normal protein (about 0.7 g/ Kg/day) and high fat content (enough to satisfy the patients’ caloric needs) from meals prepared with common foods.

The ketogenic ratio (grams of fat/grams of carbohydrates plus proteins) of normo-caloric diets ranges from 2 to 3:1. The ketogenic ratio is not relevant for the VLEKD, since it is characterized by an extreme reduction of fats and carbohydrates, mimicking fasting that is the physiologic condition inducing ketogenesis. It is thus not calculated during the VLEKD planning. The nutraceutical supplements to the diets can be found in Table 1 of Di Lorenzo et al. 2016 (Di Lorenzo et al. 2016).

**Table 1:** Baseline clinical and demographic characteristics of migraine patients. (means ± SD).

*Characteristics*	All patients		Normo-caloric		Hypo-caloric	
Women (n)	17		9		8	
Age (years)	40.8 ± 11.7		37.8 ± 9.2		38.6 ± 11.8	
Duration of migraine history (years)	17.5 ± 9.6		15.2 ± 6.6		19.4 ± 11.5	

	Before	After 1-month KD	Before	After 1-month KD	Before	After 1-month KD

Attack frequency/month (n)	4.3 ± 2.6	1.3 ± 1.0 *	4.0 ± 2.5	1.1 ± 1.4 *	4.6 ± 2.8	1.5 ± 0.7 *
Attack duration (hours)	47.6 ± 27.7	16.8 ± 20.5 *	53.3 ± 24.1	13.0 ± 18.4 *	43.0 ± 30.7	19.9 ± 22.5 *
Body mass index (BMI)	25.8 ± 4.7	24.0 ± 4.2 *	22.0 ± 1.9	20.8 ± 1.7 *	28.8 ± 3.7	27.1 ± 3.5 *

* p < 0.01 between baseline and 1-month ketogenic diet.

Everyday urine dipstick tests were used to monitor ketogenesis in both groups. Patients recorded meals, daily weight, potential adverse events, and side effects in addition to the stick data in a headache report diary. Medical examination and laboratory blood tests (creatinine, gamma glutamic transpeptidase, lactic dehydrogenase, alkaline phosphatase, bilirubin, blood urea nitrogen, and aspartate aminotransferase) were performed at the beginning and the end of the 4-week KD.

### 2.3 Somatosensory evoked potentials (SSEPs)

Electrical stimulation of the right median nerve at the wrist using a constant current square wave pulse (0.1 ms) and a stimulus intensity adjusted at 1.5 times the motor threshold was used to elicit somatosensory evoked potentials (SSEPs); the repetition rate was 4.4 Hz. The 1st active electrode was placed ipsilaterally at Erb’s point referenced to the contralateral side. The 5th cervical spinous process (Cv5) and the contralateral parietal area (C3′, 2 cm posterior to C3 in the international 10–20 system) were the locations of the 2nd and 3rd recording electrodes, respectively; both were referenced to the Fz. The ground electrode was on the left arm. Digitimer^TM^ D360 pre-amplifiers (band-pass 0.05–2000 Hz, gain 1000) were used to amplify the SSEP signals, and the CED^TM^ power 1401 device (Cambridge Electronic Design Ltd, Cambridge, UK) to record the signals. During the recordings, the subjects were sitting in a comfortable armchair with both eyes open. They were instructed to focus solely on the stimulus-induced movement of their thumbs. Five hundred consecutive sweeps of 50 ms were sampled at 5000 Hz.

### 2.4 High-frequency oscillations (HFOs)

**Fig. 1:**
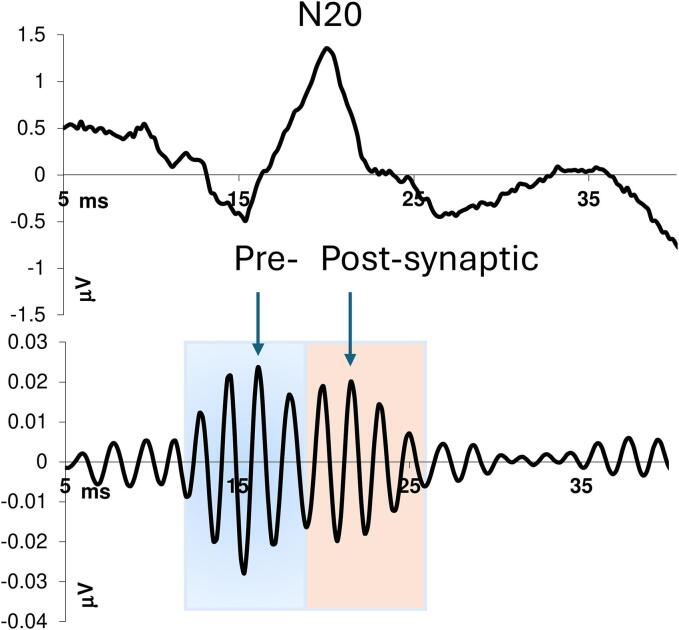
An example of somatosensory evoked potential (SSEP) data processing in a healthy subject. A broad-band SSEP recording with the N20 peak is shown in the upper panel. In the lower panel, the same recording is shown after applying a 450–750 Hz digital band-pass filter. This process makes it possible to study the high-frequency oscillations (HFOs) and particularly the pre-synaptic oscillation burst (in light blue), which reflects thalamocortical activity, and the post-synaptic burst (in pink), which reflects the activity of the primary parietal cortex. For both HFO bursts, we analysed its duration, latency and amplitude of the negative oscillatory maximum (arrow), number of negative peaks, and the intra-burst frequency.

Off-line digital zero-phase shift band-pass filtering (Barlett-Hanning window, 51 filter coefficients) allowed to extract the HFOs between 450 and 750 Hz embedded in the N20 left parietal component of the SSEP. We were able to detect two distinct HFO bursts in most recordings: an early burst in the latency interval of the conventional N20 component’s ascending slope, and a late burst in the time interval of the N20′s descending slope, which occasionally extended into the ascending slope of the N33 peak. The internal frequency of the first HFO burst was greater than that of the second burst, and there was a noticeable drop in both frequency and amplitude between the two bursts, that allowed distinguishing between them. In recordings where this distinction was not clear, we considered HFOs that occurred before the N20 peak as the first burst and those that occurred after N20 as the second burst. Latency of the negative oscillatory maximum, intra-burst frequency, number of negative peaks, and maximum peak-to-peak amplitude were measured after the stimulus artifact was eliminated (Gobbelé et al. 1998). A separate set of measurements was made for each of the two HFO bursts (Coppola et al. 2005) (Fig. 1).

### 2.5 Statistical analysis

An independent operator carried out the statistical analyses (CDL). We had anticipated a sample size of 20 patients based on prior research (Di Lorenzo et al., 2016, Di Lorenzo et al., 2019a). For all statistical analyses, SPSS for Windows, version 21, was used. On the Kolmogorov-Smirnov test, electrophysiological parameters displayed a normal distribution. To compare the clinical and electrophysiological data before and after KD, paired *t* tests were employed. In addition, as an exploratory analysis, we subanalysed patients into those with a normo-caloric diet and those with a hypocaloric diet and compared electrophysiological variables before and after 1 month of KD.

Additionally, we computed the % change in monthly frequency of attacks, BMI, and pre- and post-synaptic HFO latency of the oscillatory maximum at one month, relative to baseline. By using the Pearson test, the percentage changes in electrophysiological parameters were correlated to the percentage changes in clinical variables.

A significant p-value of 0.05 was selected for all inferential statistics.

**Fig. 2:**
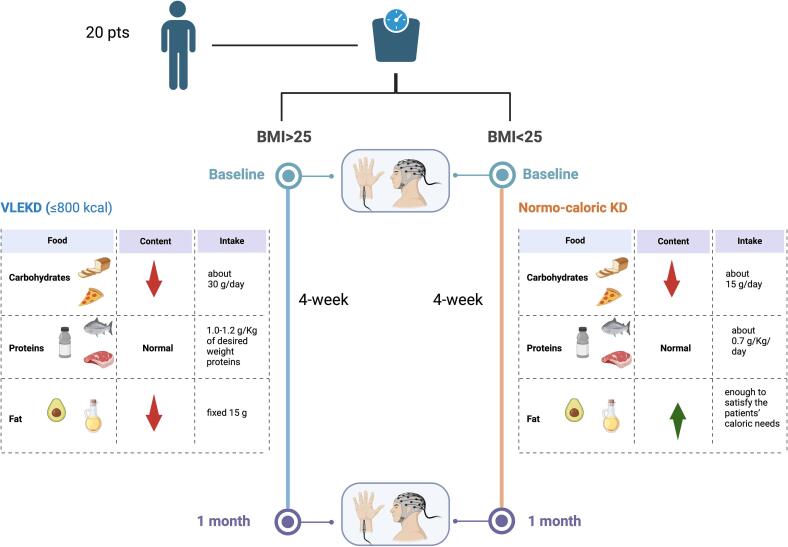
Flow chart of the study methodology. The BMI of the patients was assessed at baseline (screening visit). Patients with a BMI > 25 were assigned to a very low ketogenic diet (VLEKD) with a total of ≤ 800 kcal). The VLEKD (in blue) consisted of low carbohydrate (about 30 g/day carbohydrates), low fat (fixed 15 g lipids), and normal protein content (1.0–1.2 g/kg of desired weight proteins). Patients with a BMI < 25 were assigned to a normo-caloric KD with a total of kcal according to their needs. The normo-caloric KD (in orange) consisted of low carbohydrates (about 15 g/day), normal protein (about 0.7 g/ Kg/day), and high-fat content (enough to satisfy the patients’ caloric needs) from meals prepared with common foods. Somatosensory evoked potentials (SSEPs) were recorded in both groups at baseline (during the screening visit) and after four weeks of the respective ketogenic diet Created with BioRender.com).

All the methods are summarized in Fig. 2.

## 3 Results

Following 1-month (T1) of KD, we observed a clinical improvement with reduction of both attack frequency (from 4.3 ± 2.6 to 1.3 ± 1.0 attacks/month, t = 4.98, p < 0.001), and attack duration (from 47.6 ± 27.7 to 16.8 ± 20.5 h, t = 4.79, p < 0.001) (Table 1). At the same time, the diet had a weight-loss effect with a significant BMI decrease (from 25.8 ± 4.7 to 24.0 ± 4.2; t = 10.145, p < 0.001).

After 1-month of KD, we observed no change in the latencies and amplitudes of the N9, N13, and N20 broad-band components (all p > 0.05) (Table 2).

**Table 2:** Parameters of somatosensory-evoked potentials before and after a 1-month ketogenic diet in migraine patients (means ± SD).

**Electrophysiological parameter**	**Before**	**After**
**N9 latency (msec)**	9.85 ± 0.92	9.94 ± 0.58
**N9 amplitude (μV)**	2.95 ± 1.72	3.00 ± 1.85
**N13 latency (msec)**	13.42 ± 0.92	13.47 ± 0.89
**N13 amplitude (μV)**	1.79 ± 0.57	1.73 ± 0.46
**N20 latency (msec)**	19.12 ± 1.25	19.12 ± 1.15
**N20 amplitude (μV)**	1.79 ± 0.65	1.53 ± 0.83

**Fig. 3:**
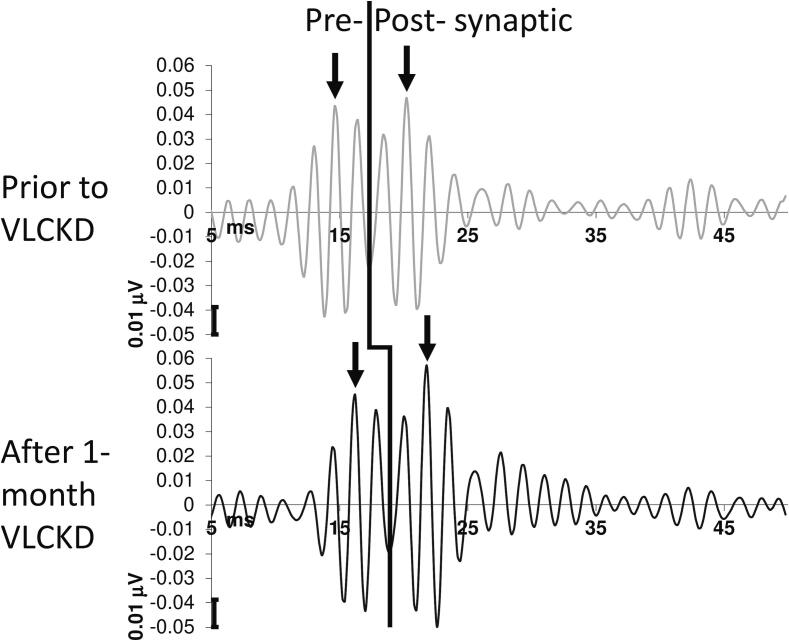
Illustrative recordings of pre- and post-synaptic somatosensory evoked high-frequency oscillations (HFOs) in a migraine patient before and after 1-month of very low-calorie ketogenic diet (VLEKD).

In comparison to T0, 1-month of KD significantly increased the latency of the negative oscillatory maximum of the pre-synaptic (from 15.61 ± 0.94 to 16.54 ± 1.49; t = 2.70, p = 0.015) and the post-synaptic burst of HFOs (from 22.38 ± 1.92 to 24.33 ± 2.72; t = 3.08, p = 0.006) (Fig. 3, Table 3).

**Table 3:** Parameters of somatosensory-evoked high-frequency oscillations (HFOs) before and after a 1-month ketogenic diet in migraine patients (means ± SD).

**Electrophysiological parameter**	**Pre-synaptic HFOs**		**Post-synaptic HFOs**	
	**Before**	**After**	**Before**	**After**
**Latency (msec)**	16.01 ± 1.52	16.67 ± 1.57 *	22.91 ± 2.40	24.54 ± 2.71 *
**Amplitude (μV)**	0.050 ± 0.028	0.049 ± 0.026	0.041 ± 0.028	0.040 ± 0.025
**Number of Peaks (n)**	3.31 ± 0.82	3.75 ± 0.91	3.95 ± 0.85	4.10 ± 0.91
**Burst durations (msec)**	5.77 ± 1.43	6.24 ± 1.58	6.54 ± 1.41	6.88 ± 1.35
**Intra-burst frequency (Hz)**	703.01 ± 55.48	701.74 ± 64.72	696.86 ± 53.87	706.38 ± 44.59

* = p < 0.01 before vs. after 1-month KD.

By contrast, no difference was found between the T0 and T1 recordings of KD for the following parameters of both pre-synaptic and post-synaptic HFOs: intra-burst frequency, number of negative peaks, maximum peak-to-peak amplitude.

In the exploratory subgroup analysis of patients, only those who followed the hypocaloric diet showed a significant delay in the latency of negative peak maximum of both pre- (t = 2.929, p = 0.017) and post-synaptic (t = 3.373, p = 0.008) HFOs. The other variables of HFOs did not vary significantly (Table 3).

### 3.1 Correlations

No correlation was found between the changes in clinical and electrophysiological variables before and after 1 month of the diet.

Only one month after the diet’s initiation (r = 0.614, p = 0.004), but not before (r = 0.346, p = 0.06), we found a positive correlation between the pre-synaptic and the post-synaptic latencies of HFO negative oscillatory maximum and number of peaks.

## 4 Discussion

This study showed that one month of KD therapy can interfere with both thalamocortical (pre-synaptic HFO burst) and cortical activity (post-synaptic HFO burst) in subjects with migraine. The KD delayed the negative maximum peak latency at the cortical and thalamocortical levels without having a short-term impact on the generation of high-frequency neuronal activity. The number of HFO peaks, the amplitude of the signal and its frequency were all unaffected after one-month of KD. Exploratory subgroup analysis of patients showed that significance is restricted to the subgroup of patients who followed a hypo-caloric instead of a normo-caloric diet, although, as previously shown with both types of diets, the energy intake seems irrelevant for the normalization of the habituation deficit of SSEPs or VEPs (Di Lorenzo et al. 2016). Indeed, a trend emerges within the latter group, suggesting that it may have achieved statistical significance with a larger sample size. We also confirm our previous clinical findings that migraine features significantly improve with the ketogenic diet, in parallel to weight-loss (Di Lorenzo et al., 2013, Di Lorenzo et al., 2015, 2019b). Of note in this study, the effects of KD on electrophysiological parameters are independent of migraine improvements.

The exact mechanism by which the ketogenic diet exerts its clinical effects and the exact level of action in the central nervous system is still under debate (see (Di Lorenzo et al. 2021)). One of the most well-established pathways for mediating the short-term preventative benefits of KD on migraine is its modulation of the glutamate/GABA balance. Indeed, KD increases inhibitory (fast GABAergic) and decreases excitatory (glutamate) neurotransmission (Nylen et al., 2008, Simeone et al., 2014), both effects considered to play a pivotal role in the anticonvulsant effect of this diet (Wheless, 2008, Klein et al., 2014). Therefore, a change in the patients' interictal aberrant information processing in migraine may be related to the brain's excitatory/inhibitory balance due to the transition from glucose to ketones as an energy substrate. One-month KD, without altering the basic level of cortical excitability, acts at the cortical level normalizing the interictally deficient visual and somatosensory non-painful evoked potentials (SSEPs) delayed habituation (Di Lorenzo et al. 2016). Additionally, the effects of KD are not limited to the non-painful sensory system, but it influences the pain-related evoked potentials normalizing the interictal cortical habituation (Di Lorenzo et al. 2019a). Of note, even if the above mentioned deficit of habituation of multimodal cortical evoked potentials are normalized by KD, a similar effect is not exerted at the brainstem level, since KD does not normalize the deficit of habituation of blink reflex. Based on these findings, we speculated that the cerebral cortex may be the main location of KD-related modulation (Di Lorenzo et al. 2019a), which could also explain the recently observed improvement in sleep quality after KD in overweight subjects (Barrea et al. 2023) and in migraine patients (Merlino et al. 2023).

According to our present findings, KD significantly increases the latency of the negative oscillatory maximum in the pre-synaptic HFO (thalamocortical activity), and post-synaptic HFO (cortical activity). Other HFO parameters, such as amplitude, duration, and number of oscillations, were not affected. In animal models it has been shown that HFOs are electrophysiological markers for cortical spike bursts (Baker et al. 2003). The high-frequency bursts superimposed on the conventional broad-band somatosensory response are originally thought to reflect rapidly repeating population spikes in thalamo-cortical axons, with additional wavelet generators within the grey matter of S1 (Curio 2000). In our case, both the thalamocortical drive and primary somatosensory cortical − high-frequency impulse propagation, indexed by peak latencies, can be modified by a single month of KD.

To explain these results, we can claim the abovementioned mechanisms of KD interferencing with the glutamate/GABA balance in migraine.

Within an excitatory-inhibitory loop, the GABA_A-B_ receptors are essential for controlling synaptic transmission and plasticity in the thalamus and cortex. Pharmacological blockage of GABA_B_ receptors, for instance, may disrupt pathways involved in long-term potentiation (Sanchez-Vives et al. 2021). Presynaptic GABA_B_ receptors block glutamate release at the cortical level and in thalamocortical fibers (Jia et al. 2004). All of these evidences are relevant to our findings because higher inhibition and lower excitation may result in an increased spike latency of rhythmic activity (Economo and White 2012). In accordance with our findings, it was found that lorazepam, a GABA-_A_ergic transmission enhancer, delayed the latency of the HFO peaks, with a major effect on post-synaptic peaks that were more delayed than the early pre-synaptic peaks (Haueisen et al. 2000). It is important to underscore that weight loss did not determine these electrophysiological changes, as the latter did not correlate with the percentage decrease in BMI.

In a network of neurons, the small initial delays would add up, and the subsequent oscillation peaks would be influenced by the delay of all previous oscillations. This could explain the mechanism behind the correlation between the pre- and post-synaptic latencies observed after KD. This mechanism has been previously used to explain subcortical/cortical differential latency peaks results with varying stimulus parameters (Emori et al. 1991) and after benzodiazepine administration (Haueisen et al. 2000). In such a thalamo-cortical network, the initial delay could start from neurons in the thalamus, if thalamocortical projection neurons are responsible for generating the high-frequency oscillatory activity. Alternatively, the loop could be entirely cortical if the HFOs are, for example, generated by modulations of retropropagating action potentials in pyramidal neurons (Rapp et al. 1996), which in turn reduces the latency of the subcortical relay.

Another factor that could explain the latency increase of HFO bursts would be a conduction delay in the afferent somato-sensory pathway. The fact that the latency of the broad band N20 component is not increased, however, does not favor this hypothesis (Di Lorenzo et al. 2016).

Interestingly, in the subgroup analysis, HFOs’ latencies were increased only in patients who followed a hypo-caloric KD, but not in those who followed a normocaloric KD. Experimental evidence suggests that dietary caloric restriction benefits brain functioning and may provide protection against neurodegenerative disorders (Gillette-Guyonnet and Vellas 2008). In animal models, caloric restriction has an important antiepileptic effect, irrespective of ketogenesis: seizures could no longer be experimentally induced in animals with a low-calorie diet (Greene et al., 2001, Hartman et al., 2010). Also in humans, in the pre-KD era, caloric restriction was found effective to prevent seizures (Bailey et al., 2005, Höhn et al., 2019 May). One of the possible mechanisms for this anti-convulsant effect seems to be an increase in the central bioavailability of GABA, like in fasting (Violante et al., 2009, Cabral, 2017). The neuroprotective effects of caloric restriction may also be mediated through the regulation of astrocyte functions, which are actively involved in neural plasticity and neuroprotection (Ribeiro et al. 2009). Astrocytes have the capacity to enhance glutamate uptake, glutamine synthetase activity, and glutamate decarboxylase expression in animal models, consequently leading to an augmentation in GABA production (Cheng et al., 2004, Yudkoff et al., 2006). All these mechanisms are pertinent to our findings, as, in a genetic rat model of hypermotor epilepsy, HFOs stimulate astroglial glutamate release in cortical and thalamic neurons (Fukuyama and Okada 2022). Whether this effect might be inhibited by a caloric restriction-induced slowing of HFOs would be of interest in future studies.

It remains to be shown in larger studies if low-calorie intake and KD have an additive clinical and electrophysiological effect. This was not found in a recent study by Tereshko et al. 2023 where VLEKD, normocaloric KD and low glycemic-index diet had similar beneficial effects on disability, fatigue and attack frequency in chronic and high frequency episodic migraine (Tereshko et al. 2023b). Whatever the influence of the diets on thalamocortical drive might be, we have shown here that the clinical and the electrophysiological effects can be dissociated. Nonetheless, in our study, the clinical effect seems numerically superior in the VLEKD group (Table 2). On the other hand, there is also a numerical, though non significant, increase of 1st and 2nd HFO bursts amplitudes after normocaloric KD (Table 4). One cannot exclude that these changes might become significant in future studies of a larger number of patients, which would pave the way for a wider application of VLEKD in adult neurological disorders (Cervenka et al. 2021).

**Table 4:** Parameters of somatosensory-evoked high-frequency oscillations (HFOs) in patients following a normo-caloric or an hypo-caloric ketogenic diet (KD), before and after a 1 month (means ± SD).

**Electrophysiological parameter**	**Pre-synaptic HFOs**		**Post-synaptic HFOs**	
	**Before**	**After**	**Before**	**After**
	Normo-caloric KD (N = 9)			
**Latency (msec)**	15.57 ± 0.92	16.48 ± 1.77	22.91 ± 2.33	24.16 ± 2.83
**Amplitude (μV)**	0.038 ± 0.015	0.048 ± 0.022	0.033 ± 0.014	0.040 ± 0.024
**Number of Peaks (n)**	3.33 ± 0.71	3.89 ± 1.05	3.78 ± 1.09	3.78 ± 0.83
**Burst durations (msec)**	5.74 ± 1.03	6.35 ± 1.82	6.15 ± 1.65	6.40 ± 1.04
**Intra-burst frequency (Hz)**	712.79 ± 56.34	695.39 ± 44.32	704.20 ± 43.28	709.21 ± 53.76

	Hypo-caloric KD (N = 11)			
**Latency (msec)**	15.65 ± 1.00	16.60 ± 1.30 *	21.91 ± 1.44	24.65 ± 2.47 *
**Amplitude (μV)**	0.053 ± 0.040	0.050 ± 0.032	0.052 ± 0.025	0.041 ± 0.028
**Number of Peaks (n)**	3.30 ± 0.95	3.70 ± 0.82	4.10 ± 0.57	4.30 ± 0.95
**Burst durations (msec)**	5.79 ± 1.77	6.24 ± 1.48	6.89 ± 1.12	7.23 ± 1.59
**Intra-burst frequency (Hz)**	694.21 ± 56.14	700.28 ± 80.52	690.25 ± 63.53	697.12 ± 32.03

* = p < 0.01 before vs. after 1-month KD.

We have to acknowledge several limitations to our study. First, the number of patients included was rather low and particularly that of the two normo-caloric and low-caloric subgroups. As mentioned, the difference observed between the two subgroups need to be verified on a larger sample size. Second, the diets were administered for only 1 month, which leaves open the question whether longer treatment periods might produce a different electrophysiological effect, knowing that the beneficial therapeutic effect persists up to 3 months (Tereshko et al. 2023b).

## 5 Conclusions

Our findings confirm that short-term KD decreases disability in migraine patients and support the hypothesis that it modifies the central excitation/inhibition balance, as suggested by the delayed transmission of impulses across the thalamo-cortical network. However, the electrophysiological change was observed in VLEKD and not in normocaloric KD, indicating that low calorie intake is crucial in its production. It remains to be determined if the slowing of SSEP thalamo-cortical activity is related to the improvement in the SSEP habituation deficit we have previously reported after VLEKD.

## 6 Financial support

This research did not receive any specific grant from funding agencies in the public, commercial, or not-for-profit sectors.

## Authors contributions

CA & GS made substantial contributions to interpretation of data as well as in drafting the manuscript. FC, MS, GC, and CDL were implied in the interpretation of data as well as in drafting the manuscript; JS gave critical revision of the manuscript for important intellectual content. GS, CA, and FC were implied in recording and analyzing data. ADR was implied in statistical analysis. VP and EC were implied in neuroophthalmological analysis. All authors read and approved the final manuscript.

## Declaration of Competing Interest

The authors declare that they have no known competing financial interests or personal relationships that could have appeared to influence the work reported in this paper.
